# Mechanism and Consequences of The Impaired Hif-1α Response to Hypoxia in Human Proximal Tubular HK-2 Cells Exposed to High Glucose

**DOI:** 10.1038/s41598-019-52310-6

**Published:** 2019-11-01

**Authors:** Coral García-Pastor, Selma Benito-Martínez, Victoria Moreno-Manzano, Ana B. Fernández-Martínez, Francisco Javier Lucio-Cazaña

**Affiliations:** 1 Departamento de Biología de Sistemas, Universidad de Alcalá, Alcalá de Henares, Madrid, Spain; 2 Neuronal and Tissue Regeneration Laboratory, Centro de Investigación Príncipe Felipe, Valencia, Spain; 3 Departamento de Biología, Universidad Autónoma de Madrid, Madrid, Spain

**Keywords:** Transcription factors, Mechanisms of disease, Ubiquitylation

## Abstract

Renal hypoxia and loss of proximal tubular cells (PTC) are relevant in diabetic nephropathy. Hypoxia inhibits hypoxia-inducible factor-1α (HIF-1α) degradation, which leads to cellular adaptive responses through HIF-1-dependent activation of gene hypoxia-responsive elements (HRE). However, the diabetic microenvironment represses the HIF-1/HRE response in PTC. Here we studied the mechanism and consequences of impaired HIF-1α regulation in human proximal tubular HK-2 cells incubated in hyperglycemia. Inhibition at different levels of the canonical pathway of HIF-1α degradation did not activate the HIF-1/HRE response under hyperglycemia, except when proteasome was inhibited. Further studies suggested that hyperglycemia disrupts the interaction of HIF-1α with Hsp90, a known cause of proteasomal degradation of HIF-1α. Impaired HIF-1α regulation in cells exposed to hyperglycemic, hypoxic diabetic-like milieu led to diminished production of vascular endothelial growth factor-A and inhibition of cell migration (responses respectively involved in tubular protection and repair). These effects, as well as impaired HIF-1α regulation, were reproduced in normoglycemia in HK-2 cells incubated with microparticles released by HK-2 cells exposed to diabetic-like milieu. In summary, these results highlight the role of proteasome-dependent mechanisms of HIF-1α degradation on diabetes-induced HK-2 cells dysfunction and suggest that cell-derived microparticles may mediate negative effects of the diabetic milieu on PTC.

## Introduction

Diabetic nephropathy (DN) is the major cause of end-stage renal disease. It is now evident that functional and structural changes in proximal tubular cells (PTC) play a critical role in the development of the disease^1^. Although a high glucose (HG) microenvironment is the primary causative factor for the development of DN, hypoxia also plays a relevant role in the development and progression of DN^2,3^.

The renal adaptive responses to combat hypoxia are mediated by transcription factor hypoxia-inducible factor-1 (HIF-1). The regulatory mechanisms of HIF-1 and its role in hypoxia-induced cell responses to hypoxia have been respectively recently reviewed^4,5^. HIF-1 is composed of a HIF-1β subunit, which is constitutively produced, and a labile HIF-1α subunit, which is quickly degraded in the presence of oxygen through hydroxylation in specific proline residues by a series of 2-oxoglutarate and Fe^2+^ dependent dioxygenases (prolyl hydroxylases, PHDs). HIF-1α binds thereby to tumour suppressor von Hippel-Lindau (pvHL) protein, which mediates the ubiquitination and degradation of HIF-1α in the proteasome. In hypoxia, HIF-1α protein accumulates rapidly -in the absence of a significant increase in HIF-1α mRNA- and dimerizes with the β subunit because HIF-1α is stabilized by inhibition of the canonical PHD-pvHL-proteasome degradation pathway. This heterodimer, together with transcriptional coactivators p300/CBP, binds then to hypoxia-responsive elements (HRE) in target genes thereby activating the transcription of genes involved in adaptative and survival responses to hypoxia (i.e. angiogenesis, cell proliferation, anaerobic metabolism, wound healing, etc). HIF-1α cell levels are also regulated by oxygen-independent mechanisms such are growth factor signalling pathways, Mdm-2 (mouse double minute 2 homolog)-mediated ubiquitin-proteasome pathway and binding to heat shock protein 90 (Hsp90) among others^6^.

Repression of the HIF-1 response by HG has been specifically demonstrated in cultured PTC^7,8^. Besides the fact that inhibition of the HIF-1 pathway renders the insufficient activation of HIF-1 at the tubular compartment may be in addition pathologically relevant in the hypoxic diabetic kidney because: (i) a reduction in the HIF-1-dependent production of vascular endothelial growth factor (VEGF) in DN might contribute to hypoxic tubular damage through renal ischemia^9^ by diminishing peritubular capillary density and (ii) the impaired tubular regulation of HIF-1-responsive genes might be also a key molecular pathway for inefficient kidney repair in DN: tubular cell loss is one of the features of DN^10^ and HIF-1 has been shown to regulate cell proliferation^11^ and migration^12^, which are critical responses to re-establish an intact epithelium. In the hyperglycemic/hypoxic diabetic (HGHD) microenvironment these responses are likely hampered, this resulting in an inadequate process of tubular wound healing^13^ that could be improved by targeting the abnormal regulation of HIF-1 in the diabetic milieu.

In addition to the direct effect of the HGHD milieu on kidney cells, cell-derived microparticles (MPs) also contribute to DN onset and progression (reviewed by Lu *et al*., 2017^14^). MPs are originated by budding of cell surface and shift of phosphatidylserine from the inner to the outer leaflet of the cell membrane. This allows for detection of MPs because phosphatidylserine binds annexin V^14^. MPs play an important role in intercellular communication and they are distinguishable from other types of cell-derived extracellular vesicles (i.e. exosomes and apoptotic bodies) according to the size, mechanism of formation, surface markers and content. Exosomes (30–120 nm) are smaller than MPs (40–1000 nm) and apoptotic bodies (>1 µm), which are the largest extracellular vesicles^15^. All the cells along the nephron release MPs that can be detected in human urine^15,16^. We have previously found that both expression of HIF-1α and HIF-1-dependent production of VEGF in human proximal tubular HK-2 cells are regulated by MPs released by vascular endothelial cells^17^. Therefore, it is conceivable that, the MPs produced by HK-2 cells in the HGHD microenvironment may convey signals that contribute to impair the regulation of HIF-1 in these cells.

Despite the pathological relevance for DN of the insufficient activation of HIF-1, the detailed mechanisms underlying impairment of HIF-1 pathway at the tubular compartment remain poorly understood. Here we have studied in human proximal tubular HK-2 cells exposed to diabetic-like milieu (i.e. hyperglycemic/hypoxic conditions) the mechanisms responsible for the impaired HIF-1α up-regulation, the consequences of the defective HIF-1α up-regulation on cell responses involved in tubular protection and repair and the role of cell-derived MPs as vectors of intercellular communication that might mediate HIF-1α-related effects of the diabetic milieu.

## Results

### Impaired activation of the canonical HIF-1α-HRE pathway in HK-2 cells exposed to high glucose

Impaired activation of the HIF-1α-HRE pathway in the presence of high glucose (HG) has been specifically demonstrated in hypoxic PTC but the mechanisms involved remain unclear. In order to address this issue, we first studied the response under hyperglycemia of the HIF-1α-HRE pathway in HK-2 immortalized PTC cells in which the canonical oxygen-, iron-PHD-pVHL-ubiquitin-dependent proteasome pathway of HIF-1α degradation had been inhibited at different levels.

**Figure 1:**
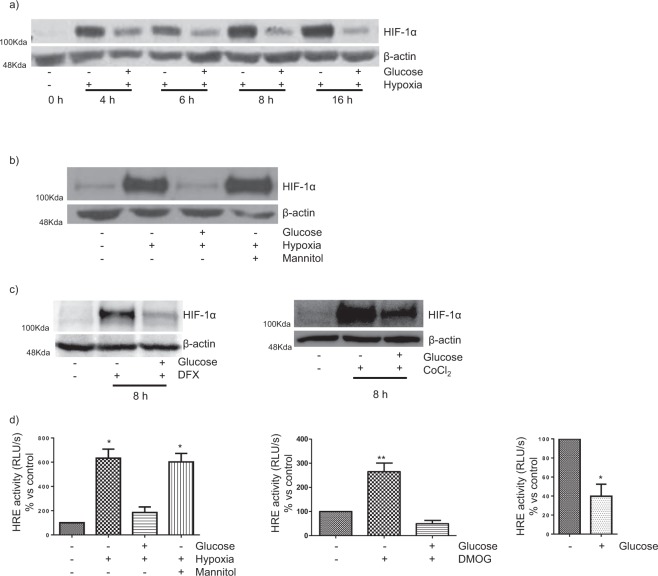
Impaired activation of the canonical HIF-1α-HRE pathway in human PTC in diabetic-like milieu. (**a**) High glucose inhibits the accumulation of HIF-1α in hypoxia as assessed by Western blot analysis. (**b**) Hypoxia-induced increase in HIF-1α expression is not inhibited by osmotic control mannitol. (**c**) High glucose inhibits the accumulation of HIF-1α induced by hypoxia mimetic agents 380 µM desferrioxamine (DFX) or 200 mM cobalt chloride (CoCl_2_). Incubation time: 8 hours. (**d**) HG diminishes the functional activity of HIF-1α. HK-2 cells (left and center) or pvHL-defective RCC4- cells (right) were transiently transfected with a hypoxia-responsive element (HRE)-luciferase reporter construct. Then HK-2 cells were exposed to high glucose under normoxic or hypoxic conditions (left) or to 0.6 mM PHD inhibitor dimethyloxaloglycine (DMOG, center). After 16 h incubation, luciferase activity in cell lysates was measured and expressed as Relative Luminescence Units (RLU/s). Each bar represents the luciferase activity normalized by Renilla luciferase activity and expressed as the mean ± SD of three independent experiments. Statistical analysis: Left: *P < 0.001 vs control and hypoxia/HG; Center: **P <0.001 vs other groups; Right: *P < 0.05 vs control. General information: HK-2 cells were grown in 5.5 mM glucose (low glucose). In the experiments, cells were exposed to low glucose (glucose -) or high glucose (glucose + : 25 mM glucose final concentration) or osmotic control (19.5 mM mannitol plus 5.5 mM glucose). When required, cells were placed in hypoxia (1% O_2_). Western blot analysis of HIF-1α: photographs are representative of the results obtained. Equal protein loading was confirmed by probing with an anti-β-actin antibody.

Under normoxic conditions, very low HIF-1α protein expression could be detected by Western blot analysis of whole HK-2 cell extracts, and exposure to hypoxia for up to 16 h resulted in increased expression of HIF-1α. However, in the presence of HG, hypoxia-dependent accumulation of HIF-1α protein was impaired (Fig. 1a). The effect of HG was evident after 4 h incubation and was not reproduced by osmotic control mannitol (Fig. 1b). HG also blunted the basal expression of HIF-1α in normoxia (results are not shown). In a similar manner, exposure of HK-2 cells to hypoxia mimetic agents desferrioxamine (DFX, an iron chelator) or cobalt chloride (Cl_2_Co, a competitive inhibitor of iron which also inhibits the interaction between HIF-1α and pvHL)^18^ inhibited hypoxia-dependent accumulation of HIF-1α (Fig. 1c). Next we investigated whether the decrease of HIF-1α protein levels induced by HG is followed by a decrease in the transcriptional activation function of HIF-1α. To study this, luciferase activity was measured in cells which were transiently transfected with a construct consisting of 9 repeats of the VEGF promoter HRE upstream of a luciferase gene (9xHRE-Luc^19^). This reporter gene exhibited a strong hypoxia-dependent activation response in cells exposed to low glucose (LG) which was significantly reduced when cells were incubated with HG but not with osmotic control mannitol (Fig. 1d, left). HG also inhibited HIF-1α-dependent activation of the 9xHRE-Luc construct either after treatment of HK-2 cells with the well-characterized PHD inhibitor dimethyloxaloglycine (DMOG, Fig. 1d, center) or in pvHL-defective RCC4 cells (Fig. 1d, right).

Therefore, HG determined a reduction in the expression of HIF-1α and/or the transcriptional activation function of HIF-1α despite the fact that the canonical oxygen-, iron-PHD-pVHL-ubiquitin-dependent proteasome pathway of HIF-1α degradation had been inhibited at different levels by hypoxia, DFX, Cl_2_Co, DMOG or pVHL deficiency. This fact suggests that the canonical pathway of HIF-1α degradation is not involved in the mechanism though which HG represses the HIF-1α-HRE pathway in hypoxic HK-2 cells.

### Repression of the HIF-1α-HRE pathway in HK-2 cells by HG is independent of inhibition of HIF-1α mRNA transcription and increased reactive oxygen species (ROS) production

**Figure 2:**
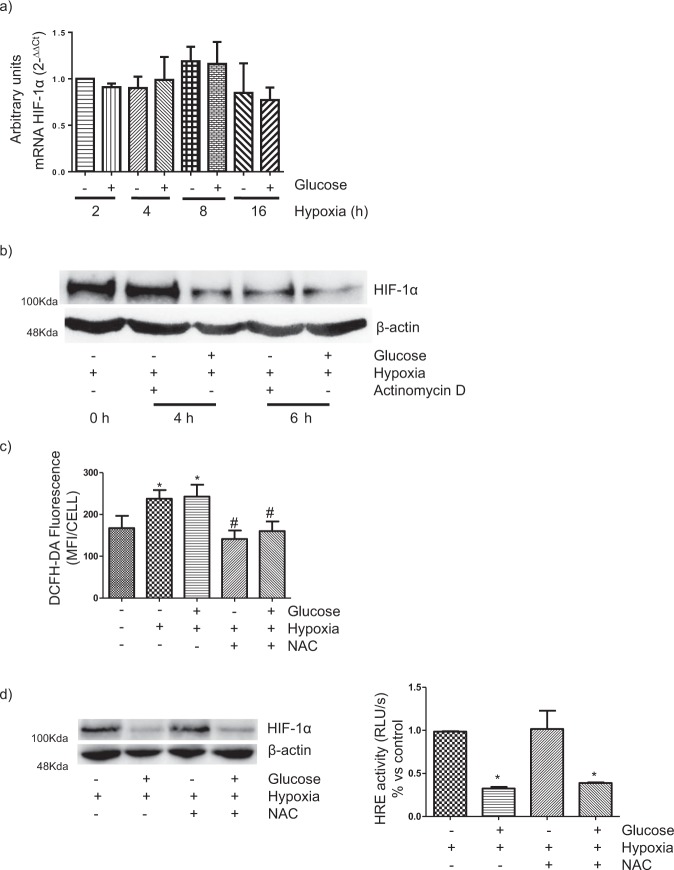
Repression of the HIF-1α-HRE pathway in human PTC in diabetic-like mileu is independent of inhibition of HIF-1α mRNA transcription and increased reactive oxygen species (ROS) production. (**a**) Expression of HIF-1α mRNA is not affected by high glucose. RT-qPCR. The relative quantification of the target gene was normalized to β-actin mRNA. There were no statistically significant differences between the different experimental groups. (**b**) High glucose reduces HIF-1α expression earlier than transcriptional inhibitor actinomycin D. HIF-1α expression was assessed by Western blot analysis. Equal protein loading was confirmed by probing with an anti-β-actin antibody. (**c**) Intracellular ROS levels increase under high glucose and hypoxia in a NAC-sensitive manner. Incubation time: 24 h. Intracellular ROS were detected with DCFH-DA and measured by flow cytometry (Mean Fluorescence Intensity/cell) *P < 0.05 vs control, ^#^P < 0.05 vs hypoxia; (**d**) NAC did not prevent the repression by high glucose of the HIF-1α-HRE pathway. Left: HIF-1α expression was assessed by Western blot analysis. Right: Cells were previously transfected transiently with a hypoxia-responsive element (HRE)-luciferase reporter construct. Luciferase activity in cell lysates was measured and expressed as Relative Luminescence Units (RLU/s). Each bar represents the luciferase activity normalized by Renilla luciferase activity and expressed as mean ± SD. Statistical analysis: *P < 0.01 vs hypoxia/low glucose and hypoxia/NAC. General information: HK-2 cells were grown in 5.5 mM glucose (low glucose). In the experiments, cells were exposed to low glucose (glucose -) or HG (glucose+: 25 mM glucose final concentration) and/or hypoxia (1% O_2_). Unless otherwise indicated, incubation time was 16 h and pre-incubation with 10 μM NAC or 1 µg/ml actinomycin D lasted one additional hour. Each experiment was performed three times (n = 3) and photographs are representative of the results obtained.

Another potential mechanism through which HG may interfere with HIF-1α up-regulation in hypoxia is targeting the transcription of HIF-1α. Therefore, we investigated the influence of HG on HIF-1α mRNA levels in hypoxia. HK-2 cells were cultured as indicated in Methods in normoxia and hypoxia with LG or HG concentrations. Then, expression of HIF-1α mRNA was analyzed Q-RT-PCR (Fig. 2a) using GADPH as internal control. Our results indicated that HG did not modify the levels of HIF-1α mRNA found in hypoxia. In another set of experiments, we compared the time-course of the decrease in HIF-1α protein expression in HK-2 cells which were incubated first in hypoxia and then exposed to HG or the inhibitor of transcription actinomycin D. As shown in Fig. 2b, after 4 h incubation, expression of HIF-1α was dramatically decreased in cells incubated in HG but not in cells incubated with the transcriptional inhibitor. Therefore, the results shown in Fig. 2a,b do not support the notion that HG inhibits HIF-1α mRNA transcription.

Oxidative stress occurs in kidneys of diabetic rats^20^ and it has been involved in the inhibition by HG of hypoxic responses in rat PTC, including these related to the HIF-1α-HRE pathway^7^. Furthermore, HG has been found to increase reactive oxygen species (ROS) in renal tubular epithelial cells^21,22^. Therefore, we decided to assess the role of ROS in the repression of the HIF-1α-HRE pathway by HG in HK-2 cells. We first estimated with the oxidative fluorescent DCFH-DA the intracellular levels of ROS in HK-2 cells which were incubated under HG/hypoxia conditions and found that they were increased in an antioxidant N-acetylcysteine (NAC)-sensitive way (Fig. 2c). We next asked whether suppression by NAC of the increased production of ROS would restore the HIF-1α-HRE response under hypoxia in HK-2 cells exposed to HG. As shown in Fig. 2d, this was not the case, which suggests that ROS are not involved in the mechanisms through which HG repress this pathway in HK-2 cells.

In summary, the results shown in Fig. 2 suggest that neither decreased HIF-1α mRNA transcription or increased ROS production contribute to the inhibitory effect of HG on the HIF-1α-HRE pathway in HK-2 cells.

### HG diminishes the stability of HIF-1α through targeting a non-canonical pathway for its proteasomal degradation. Potential role of the interaction between HIF-1α and Hsp90

**Figure 3:**
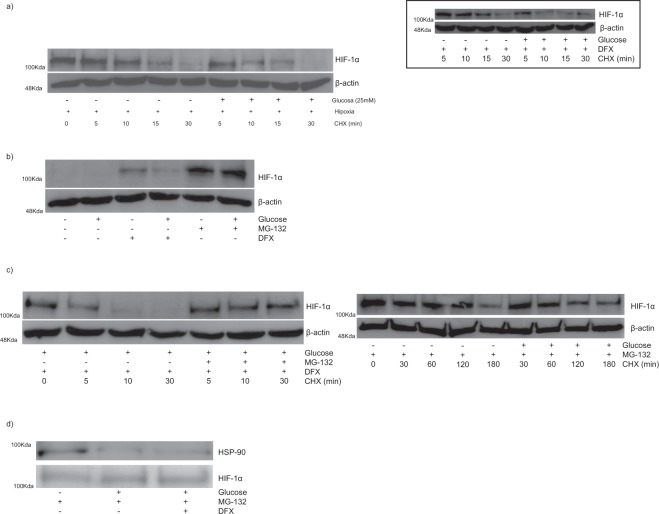
Proteasomal-dependent repression of HIF-1α up-regulation in human PTC in diabetic-like milieu: role of reduced stability of HIF-1α associated to disruption of its interaction with Hsp90. (**a**) High glucose reduces the stability of HIF-1α in cells in which the canonical oxygen-, iron-PHD-pVHL-ubiquitin-dependent proteasomal pathway of HIF-1α degradation has been inhibited. HK-2 cells were pre-incubated in low glucose under hypoxia (1% O_2_) or with desferrioxamine (DFX, inset) for 4 h. Thereafter, 50 µg/ml of the protein translation inhibitor cycloheximide (CHX) was added and cells were incubated as indicated. (**b**) Proteasome inhibitor MG-132 blocks the inhibitory effect of high glucose on DFX-induced increase in HIF-1α accumulation. Cells were incubated for 8 h with either DFX or MG132 (**c**) Proteasome inhibitor MG-132 increases the stability of HIF-1α in high glucose. Left: Cells were treated in low glucose with DFX for 4 h before being treated with CHX and MG-132 in high glucose (DFX was refreshed). Right: Cells in low glucose were pre-treated with MG-132 for 1 h. Then, medium was replaced by either low glucose or high glucose and cells were treated with MG-132 and CHX. (**d**) Interaction between HIF-1α and Hsp90 is reduced by HG. HK-2 cells in low glucose or high glucose were treated with or without DFX in the presence of MG132 for 8 h, and cell extracts were subjected to immunoprecipitation using antibodies against HIF-1α. After separation of the immunoprecipitates by electrophoresis, protein levels of HSP90 and HIF-1α were determined by Western blot analysis. General information: HK-2 cells were grown in 5.5 mM glucose (low glucose). In the experiments, cells were exposed to low glucose (glucose –) or high glucose (glucose+: 25 mM glucose final concentration). DFX and MG132 were used at 380 µM and 10 µM concentration, respectively. Western blot analysis of HIF-1α and immunoprecipitation of HIF-1α and Hsp90: photographs are representative of the results obtained.

HIF-1α accumulates in hypoxia because it is stabilized by inhibition of the canonical oxygen-, iron-PHD-pvHL-dependent proteasome pathway of HIF-1α degradation. However, the results shown in Fig. 1 do not suggest that this post-translational mechanism was involved in the inhibition by HG of hypoxia-induced HIF-1α up-regulation. Therefore we hypothesized that HG might impair HIF-1α up-regulation through inhibiting a non-canonical post-translational mechanism of HIF-1α stabilization. This hypothesis was tested in the following way: first, the canonical, oxygen-dependent pathway of HIF-1α degradation was inhibited in HK-2 cells by incubating them in hypoxia for 4 h, which resulted in robust expression of HIF-1α (Fig. 3a); then, keeping the cells in hypoxic conditions, *de novo* protein synthesis was blocked with cycloheximide (CHX), which allowed for assessing the stability of HIF-1α in cells in HG as compared to cells in LG. First of all, we will analyse the time-course of the degradation of HIF-1α in HK-2 cells in LG. As shown in Fig. 3a, HIF-1α protein levels declined quickly after treatment with CHX despite proteasomal degradation of HIF-1α through the canonical pathway was inhibited by hypoxia, which reflects the activity in HK-2 cells of proteasome-independent pathways of HIF-1α degradation^23^. This activity increased in HG, as inferred from the quicker rate of decay of the accumulated HIF-1α protein (Fig. 3a). Similar results were found when we studied the stability of HIF-1α in HK-2 cells in which accumulation of HIF-1α was achieved through inhibition with DFX of the canonical pathway of HIF-1α degradation (Fig. 3a, inset). Collectively, the results shown in Figs 1, 2a,b and 3a suggest that the inhibition by HG of the HIF-1α-HRE pathway in HK-2 cells is due to loss of HIF-1α stability through a non-canonical pathway of HIF-1α degradation

Proteasome degrades HIF-1α through ubiquitin-dependent or -independent pathways^6^. To examine the role of proteasome in the post-translational regulation of HIF-1α protein by HG, we used the proteasomal inhibitor MG-132. MG-132, unlike DFX, overcame the inhibitory effect of HG on HIF-1α accumulation (Fig. 3b) and increased notably the stability of HIF-1α under HG in DFX-treated cells (Fig. 3c, left). Furthermore, HG did not affect the decay of HIF-1α when HK-2 cells which were treated with MG-132 (Fig. 3c, right). These results indicate that proteasomal degradation of HIF-1α, most likely through a non-canonical oxygen-, PHD-pVHL-independent pathway (as stated above), is involved in the inhibitory effect of HG on HIF-1α up-regulation.

Hsp90 is a molecular chaperone which has been previously shown to be required for the stability and function of HIF-1α^24^. When the physical interaction between HIF-1α and Hsp90 is disrupted with geldanamycin in RCC4 cells lacking functional pvHL, HIF-1α is efficiently ubiquitinated, which leads to its oxygen-independent proteasome-mediated degradation^24^. Accordingly, we have previously found that geldanamycin abolishes the protection of HIF-1α from proteasomal degradation in HK-2 cells in which the PHD-pvHL-proteasomal pathway was inhibited by DFX^23^. Since HG disrupts the association of Hsp90 with eNOS^25^, we hypothesized that it might also disrupt the physical interaction between HIF-1α and Hsp90. If so, it would explain the inhibitory effect of HG on the accumulation of HIF-1α in HK-2 cells despite they had inhibited the PHD-pVHL-proteasome pathway of HIF-1α degradation (Fig. 1). The effect of HG on the interaction between HIF-1α and Hsp90 was investigated using immunoprecipitation with specific antibodies in HK-2 cells treated with both DFX (to inhibit the PHD-pvHL pathway of HIF-1α proteasomal degradation) and MG132 (to inhibit the proteasome and thereby preventing the loss of HIF-1α by HG). Our results (Fig. 3d) indicated that the interaction between HIF-1α and Hsp90 was significantly reduced by HG.

### The diabetic environment inhibits HIF-1α-regulated responses that are potentially involved in tubular protection and repair

**Figure 4:**
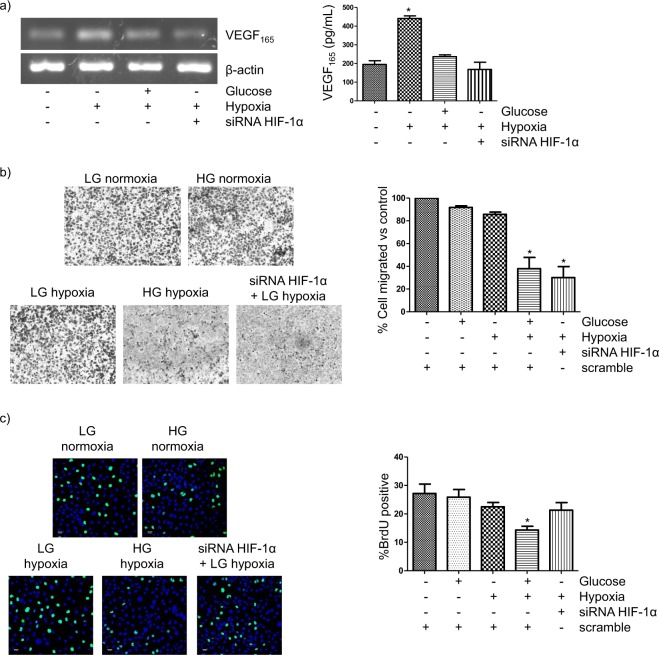
The diabetic-like milieu inhibits HIF-1α-regulated responses that are potentially involved in tubular protection and/or repair. (**a**) Hypoxia-induced increase in the expression of VEGF-A, a HIF-1α-regulated gene, is blunted by high glucose. Left. Expression of VEGF-A mRNA. HK-2 cells were transfected with either scrambled RNA or HIF-1α siRNA. 24 h later, cells were incubated for 16 h in normoxic or hypoxic conditions under either LG or HG. Semi-quantitive RT-PCR: equal mRNA loading was confirmed by assessing the β-actin gene expression level. Right: Cells were treated as in Fig. 4 a left for up to 24 h and VEGF secreted to the extracellular medium was determined by ELISA. Statistical analysis: *P < 0.001 vs other groups. (**b**) HK-2 cell migration across filters is inhibited by the combined effects of high glucose and hypoxia. Role of HIF-1α. Cells were transfected with either scrambled RNA or HIF-1α siRNA. 24 h later, a total of 250,000 cells in 0.1 ml of low glucose (LG) or high glucose (HG) medium with 0% FBS and 0% ITS were seeded in the upper chamber of each transwell insert, which was put in a 24-well plate containing LG or HG medium with 10% FBS and 1% ITS. After 24 h incubation under normoxic or hypoxic conditions, the inserts were removed and the cells that migrated to the bottom of the transwell membrane were counted after staining with 0.1% crystal violet. Statistical analysis: *P < 0.05 vs normoxia/LG, normoxia/HG and hypoxia/LG. (**c**) Proliferation of HK-2 cells is inhibited by the combined effects of HG and hypoxia in a HIF-1α-independent manner. Cells were first transfected as in b) and then seeded in 24-well plates (40 × 10^3^ cells/well) and incubated for 24 h in LG or HG under hypoxic or normoxic conditions. Cells were pulsed with 10 μM BrdU during the last 1 h of incubation and incorporation of BrdU into newly synthesized DNA strands was quantified as indicated in Methods. Statistical analysis: *P < 0.01 vs other groups. General information: HK-2 cells were grown in 5.5 mM glucose (low glucose). Then, they were transfected and used for experiments in which they were exposed to low glucose (glucose –) or high glucose (glucose + : 25 mM glucose) and/or hypoxia (1% O_2_). Each bar is the mean ± SD. The photographs are representative examples of three independent experiments (n = 3).

Adequate peritubular capillary densities, which are regulated (among others) by VEGF, are likely to be protective against hypoxic tubular damage in DN. However, because VEGF is a HIF-1-responsive cytokine, the impaired tubular regulation of HIF-1 in DN might result in impaired hypoxia-induced VEGF expression. Indeed, this is the case in immortalized rat PTC^7^ but whether HG also impairs hypoxia-induced VEGF expression in human PTC is currently unknown. In order to clarify this issue, as well as the role of HIF-1α in the expected changes in VEGF expression, VEGF mRNA and VEGF protein were respectively determined by RT-PCR and ELISA in HK-2 cells which were first transfected with either scrambled RNA or HIF-1α siRNA and then cultured under normoxia/hypoxia and LG/HG. As shown in Fig. 4a HG significantly blunted the increase induced by hypoxia in the expression of both VEGF mRNA and VEGF protein. Hypoxia-induced increase in VEGF mRNA and protein was also prevented by HIF-1α knock-down, which indicated that the inhibition of HIF-1-HRE pathway found above was responsible for the blunting effect of HG on VEGF expression.

Tubular cell loss is one of the features of DN^10^. Cell proliferation and migration are critical processes in the wound healing response that re-establishes an intact epithelium. Taking into account that HG impairs both tubular wound healing^26^ and HIF-1α regulation, and that HIF-1α is a regulator of both cell migration and proliferation^11,12^, we hypothesized that these processes might be inhibited in HK-2 cells by the diabetic environment (i.e. HG and hypoxia) in a HIF-1α-dependent manner. In order to test this hypothesis, we first studied the effects of HG and hypoxia on HK-2 cell migration. Our results indicated that neither HG nor hypoxia affected the colonization of the lower chamber of the transwell insert by HK-2 cells transfected with scrambled siRNA (Fig. 4b). However, migration of HK-2 cells was inhibited by the combined effects of HG and hypoxia, these conditions resembling the environment of PTC in the diabetic kidney. On the other hand, transfection with siRNA HIF-1α resulted in inhibition of cell migration under hypoxia in a similar degree to that found in HK-2 cells exposed to hypoxia and HG (Fig. 4b). Therefore, the suppression by HG of the HIF-1α response to hypoxia (Fig. 1) is most likely responsible for the inhibition of HK-2 cell migration found in cells in HGHD conditions.

The effects of HG and/or hypoxia on HK-2 cell proliferation (as assessed by BrdU assay), and their dependency on HIF-1α, were analysed in cells which were first transfected with either scrambled RNA or HIF-1α siRNA. In the same way than previously described for HK-2 cell migration, cells proliferation was inhibited by the combined effects of HG and hypoxia (i.e. the diabetic-like milieu) but not by HG or hypoxia themselves (Fig. 4c). However, unlike cell migration, HIF-1α knock-down did not affect the count of BrdU positive cells in any condition tested (Fig. 4c). Therefore, the suppression of the HIF-1α response by HG (Fig. 1) does not play a role in the inhibitory effect on HK-2 cell proliferation of HGHD conditions.

### Accumulation of HIF-1α and cell migration are inhibited in HK-2 cells by microparticles (MPs) released by HK-2 cells exposed to the diabetic milieu

**Figure 5:**
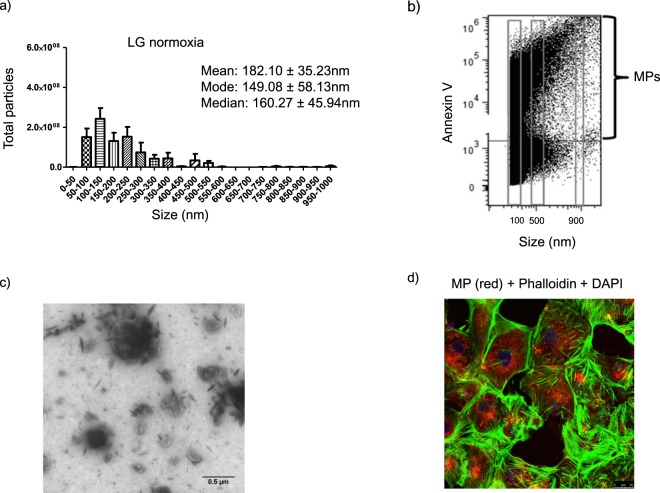
Main features of microparticles (MPs) generated by HK-2 cells. (**a**) Size distribution measured by nanoparticle tracking analysis (**b**) Detection of phosphatidylserine in MPs through staining with FITC-annexin V and flow cytometry analysis. MPs were considered as positive events of 0.3–1 μm, as assessed with calibration beads. (**c**) Transmission electron micrograph of MPs from the culture medium of HK-2 cells Original magnification:350.000. (**d**) Uptake of MPs by recipient HK-2 cells. Fluorescently prelabelled MPs (red), were incubated with HK-2 cells whose cell membrane was stained with Phalloidin (green). Uptake of MPs by HK-2 cells was checked by confocal microscopy. General information: MPs were isolated as indicated in Methods by differential centrifugation from the culture medium of 4 × 10^6^ HK-2 cells which were grown in 5.5 mM glucose. The data are typical of three separate experiments

MPs, which are produced by vesiculation of the cell plasma membrane and serve as vectors of cell-to-cell communication, have been recently involved in the pathogenesis of DN^14^. In order to evaluate the role of MPs released by HK-2 cells as vectors of the changes induced by the HGHD microenvironment in these cells, we first assessed the properties of MPs isolated by centrifugation at 18,000 xg from the culture medium of HK-2 cells grown in LG. Nanoparticle tracking analysis (Fig. 5a) showed that the mean size of MPs was 182.10 ± 35.23 nm (mean ± SD). The distribution size and mean size were similar to that previously found for MPs released by cultured human podocytes^27^. MPs were also sorted by flow cytometry and were identified as events of 0.3–1 µm with positivity for Annexin V (Fig. 5b) and they were verified to possess characteristic size and morphology by electron microscopy (Fig. 5c) and to be captured by HK-2 cells upon incubation with them (Fig. 5d). MPs internalization into HK-2 cells was not affected by the different experimental conditions. In the same the size distribution of MPs, as assessed by nanoparticle tracking analysis, was not significantly modified by any experimental condition (results are not shown).

**Figure 6:**
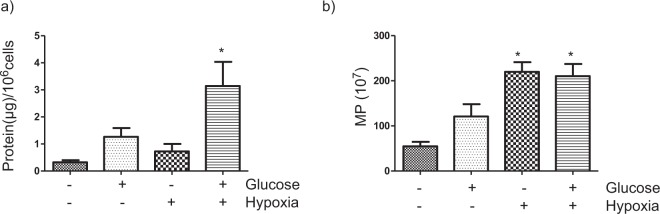
Hyperglycemic/hypoxic diabetic microenvironment increases the release of microparticles (MPs) by HK-2 cells. (**a**) MPs expressed as a function of the ratio of their protein content to the number of cells. Statistical analysis: *P < 0.001 vs other groups (**b**) MPs expressed as the number of MPs released by 2 × 10^6^ cells. Statistical analysis: *P < 0.001 vs both low and high glucose in normoxic conditions. General information: MPs were isolated from ≈2 × 10^6^ cells, as indicated in Methods, by differential centrifugation from the culture medium of HK-2 which were first grown in low glucose (5.5 mM glucose) and then incubated for 24 h with either low glucose (glucose -) or high glucose (glucose+, 25 mM glucose) under normoxic or hypoxic (1% O_2_) conditions. The number of MPs was determined by nanoparticle tracking analysis and the protein content by BCA assay. Each bar is the mean ± SD of three independent experiments (n = 3).

Regarding MP release, the results were different depending on how they were expressed: MP release was significantly higher in HK-2 cells exposed to the HGHD microenvironment as compared to the other experimental groups when MPs were quantified as total MP protein content/cell number, (Fig. 6a); when the results were expressed as number of MPs shed by 2 × 10^6^ cells, hypoxia increased the formation of MPs irrespectively of the glucose content of the incubation media (Fig. 6b). In both cases, MP release was increased by the diabetic-like milieu as compared to control conditions (i.e.: MP release under normoxic/LG conditions).

**Figure 7:**
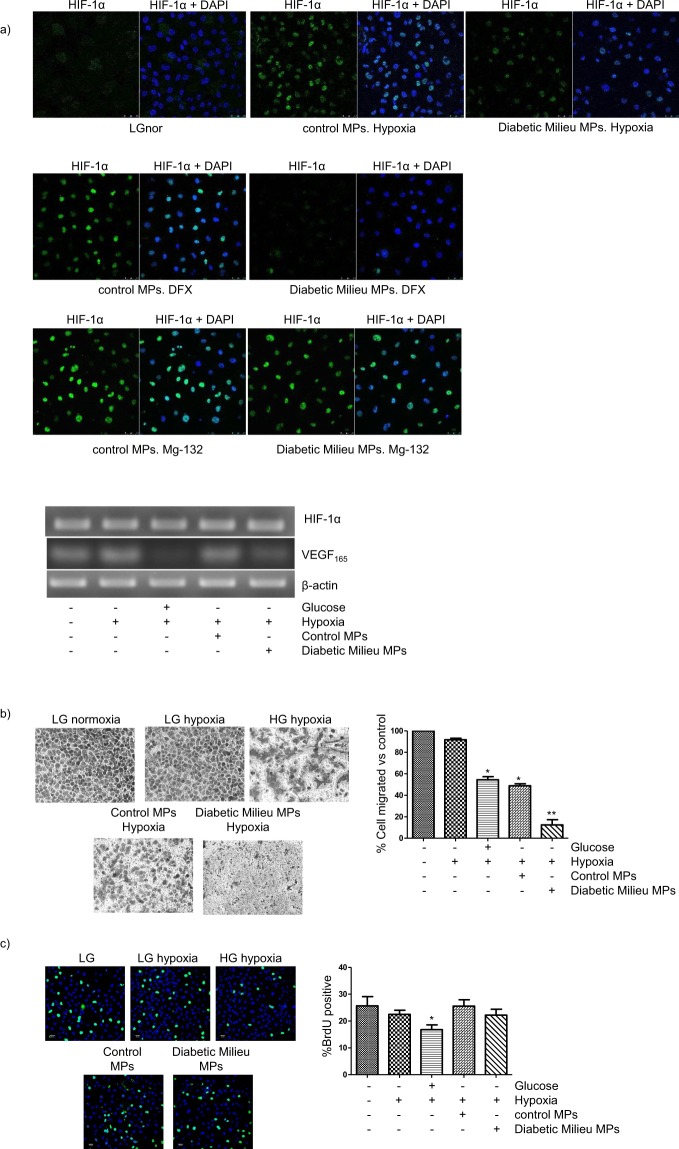
Effect of microparticles (MPs), released by HK-2 cells exposed to the diabetic milieu, on HK-2 cells. (**a**) Inhibition of the accumulation of HIF-1α in a proteasome inhibitor-sensitive manner *Upper and middle panels:* HK-2 cells were seeded in 24-well plates (40 × 10^3^ cells/well) and co-incubated in low glucose (LG) for 5 h with MPs under hypoxia (1% O_2_) or with either 380 µM DFX or 10 µM MG132. Then, expression of HIF-1α was evaluated by confocal laser scan microscopy after immunofluorescence assay with anti-HIF-1α antibody as indicated in Methods. Nuclei were stained with DAPI. Photographs are representative of the results obtained. *Lower panel:* Cells under hypoxia were incubated with MPs as above and expression of HIF-1α mRNA and VEGF-A mRNA were determined by semiquantitative RT-PCR. Equal mRNA loading was confirmed by assessing the β-actin gene expression level. (**b**) Inhibition of cell migration. HK2 cells (250.000cells/0.1 mL serum free media which contained 5.5 mM or 25 mM glucose) were seeded in the upper chambers of trans-well inserts and MPs were added if necessary. The inserts were then put in a 24-well plate containing either 5.5 mM glucose or 25 mM glucose medium with 10% FBS. After 24 h incubation under hypoxic conditions, the inserts were removed and the cells that migrated to the bottom of the transwell membrane were counted after staining with 0.1% crystal violeta. Statistical analysis: *P < 0.001 vs control, hypoxia and hypoxia plus MPs from 25 mM glucose; ** P < 0.001 vs other groups (**c**) Cell proliferation remains unaffected. HK-2 cells were seeded in 24-well plates (40 × 10^3^ cells/well) and incubated with or without MPs for 24 h in 5.5 mM or 25 mM glucose under either hypoxic or normoxic conditions. Cells were pulsed with 10 μM BrdU during the last 1 h of incubation and incorporation of BrdU into newly synthesized DNA strands was quantified as indicated in Methods. Statistical analysis: *P < 0.05 vs other groups. General information: MPs were isolated as indicated in Methods by differential centrifugation from the culture media of HK-2 cells (they were ≈ 2 × 10^6^ cells cultured in 10 mL medium) which were first grown in 5.5 mM glucose and then incubated for 24 h in hypoxia (1% O_2_) in either 5.5 mM glucose (LG, control MPs) or 25 mM glucose (HG, diabetic milieu MPs). MPs were resuspended in LG medium and added to HK-2 cells at a ratio ≈ 270,000 MPs/cell. Semi-quantitive RT-PCR for VEGF-mRNA: equal mRNA loading was confirmed by assessing the β-actin gene expression level.

We next assessed the role of MPs released by HK-2 cells as vectors of the changes induced by the HGHD-like microenvironment in these cells. We first asked whether MPs from HK-2 cells incubated in HGHD-like milieu were able to inhibit the accumulation of HIF-1α induced by hypoxia or DFX in control cells. As shown in Fig. 7a, upper panel, these MPs -but not control MPs (i.e. MPs from HK-2 cells exposed to LG under hypoxia) blunted the accumulation of HIF-1α induced by either hypoxia or DFX. Flow cytometry assessment of cell viability (annexin V/propidium iodide staining) ruled out that MPs increased HK-2 cell death (results are not shown). These results indicate that MPs are able to reproduce the inhibitory effect of the diabetic microenvironment on the accumulation of HIF-1α. In good agreement with this view, the mechanism responsible for the inhibitory effect of MPs on the accumulation of HIF-1α showed important similarities to that involved in the inhibition of HIF-1α accumulation in HGHD milieu: proteasome inhibitor MG-132 abolished the loss of HIF-1α accumulation (Fig. 7a, upper and middle panels) and there was no effect of MPs on the expression of HIF-1α mRNA (Fig. 7a, lower panel). As one might expect from the inhibition of hypoxia-induced HIF-1α, MPs generated by HK-2 cells exposed to the HGHD microenvironment also inhibited the increase in VEGF-A expression induced by hypoxia in control HK-2 cells (Fig. 7a, lower panel). In order to get additional evidence that MPs produced by HK-2 cells convey HIF-1α-dependent signals contributing to DN-related changes in PTC, we studied their effect on HK-2 cell migration (which is HIF-1α-dependent, as shown in Fig. 4b). Figure 7b shows that MPs from HK-2 cells incubated in HGHD-like milieu inhibited the migration of control cells exposed to hypoxia, being their effect even stronger than that of the HGHD microenvironment itself (probably because control MPs also inhibited cell migration). Of note, MPs did not inhibit the proliferation of HK-2 cells (Fig. 7c), probably because it is unaffected by HIF-1α (Fig. 4c).

**Figure 8:**
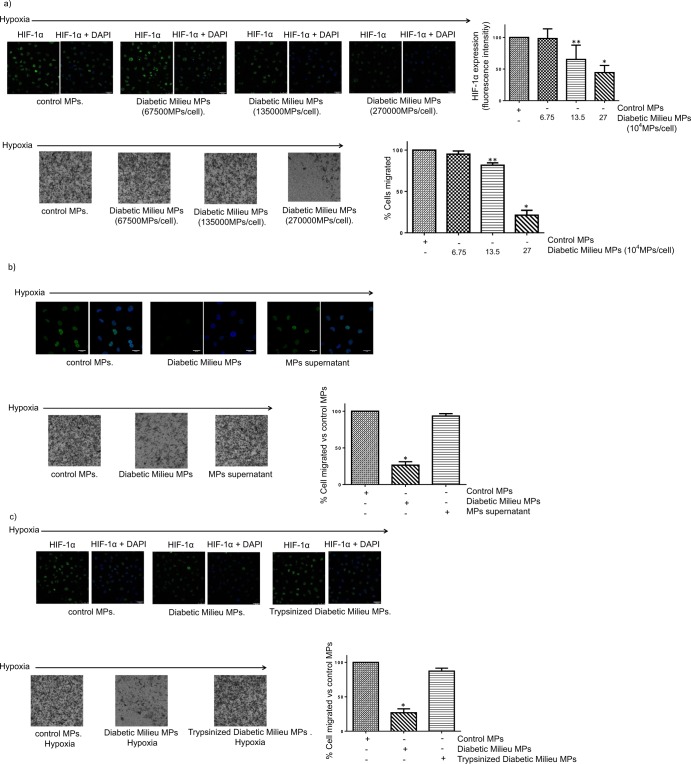
Confirmation that MPs’ biological activity is responsible for the effects of MPs suspensions: (**a**) Inhibition of the accumulation of HIF-1α (upper panel) and cell migration (lower panel) is dose-dependent: *Upper panel* HK-2 cells were seeded in 24-well plates (40 × 10^3^ cells/well) and co-incubated in low glucose medium (LG) for 5 h with MPs under hypoxia (1% O_2_). Then, expression of HIF-1α was evaluated by confocal laser scan microscopy after immunofluorescence assay with anti-HIF-1α antibody as indicated in Methods. Nuclei were stained with DAPI. Photographs are representative of the results obtained. *Lower panel* HK2 cells (250.000 cells/0.1 mL serum free LG medium were seeded in the upper chambers of trans-well inserts and MPs were added. The inserts were then put in a 24-well plate containing LG medium with 10% FBS. After 24 h incubation under hypoxic conditions, the inserts were removed and the cells that migrated to the bottom of the transwell membrane were counted after staining with 0.1% crystal violeta. (**b**) Depletion in MPs and exosomes resulted in loss of activity of MPs preparations. HK-2 cells were seeded and incubated as in a) but in addition the effect of the supernatant of MPs preparations (obtained by centrifugation at 100,000 × g/70 minutes) was also tested. (**c**) The activity of MPs is lost after incubation with trypsin. MPs were incubated for 5 min with trypsin and then with a trypsin inhibitor to stop its activity. Then, the effect of MPs on HIF-1α and cell migration was analyzed as in a). General information: MPs were isolated as indicated in Methods by differential centrifugation from the culture media of HK-2 cells (they were ≈ 2 × 10^6^ cells cultured in 10 mL medium) which were first grown in 5.5 mM glucose and then incubated for 24 h in hypoxia (1% O_2_) in either 5.5 mM glucose (LG, control MPs) or 25 mM glucose (HG, diabetic milieu MPs). MPs were resuspended in LG medium and added to HK-2 cells at a ratio ≈ 67,500 MPs/cell, 135,000 MPs/cell or 270,000MPs/cell for dose-response experiments or at a ratio ≈ 270,000 MPs/cell for the remaining experiments. Statistical analysis: *P < 0.001 vs other groups **P < 0.05 vs control MPs and 6.75 × 10^4^ diabetic milieu MPs/cell

Taken together, the results shown in Figs 6 and 7 suggest that HK-2 cells in HGHD microenvironment release MPs that propagate HIF-1α-related phenotypic alterations to naïve cells. In order to confirm this view, we performed additional experiments. In the first place, we analysed the dose-function relationship though dose-response studies. As shown in Fig. 8a, exposure of control HK-2 cells to different amounts of MPs from cells incubated in HGHD-like milieu (being the maximal amount the one used for the experiments shown in Fig. 7) inhibited the accumulation of HIF-1α induced by hypoxia and cell migration in a dose-dependent manner. In the second place, we sought to demonstrate that the activity of MPs preparations was predominantly associated with MPs rather than with soluble mediators or other co-isolated components, we compared quantitatively the activity present in/on the MPs preparation with that present in the supernatant obtained by centrifugation (100,000 x g/70 minutes, in order to deplete the sample in MPs and exosomes) of the MPs preparation. Our results (Fig. 8b) indicated that the supernatant was devoid of the biological activity present in the MPs preparation. Finally, when MPs were incubated with trypsin to digest the adhesive proteins on their surface^17^, they lost their inhibitory effect on HIF-1α expression and cell migration (Fig. 8c), which suggests that the effects of MPs are internalization dependent. Taken together, the results shown in Fig. 8 confirm that HK-2 cells in HGHD microenvironment release MPs that propagate HIF-1α-related phenotypic alterations to naïve cells.

## Discussion

**Figure 9:**
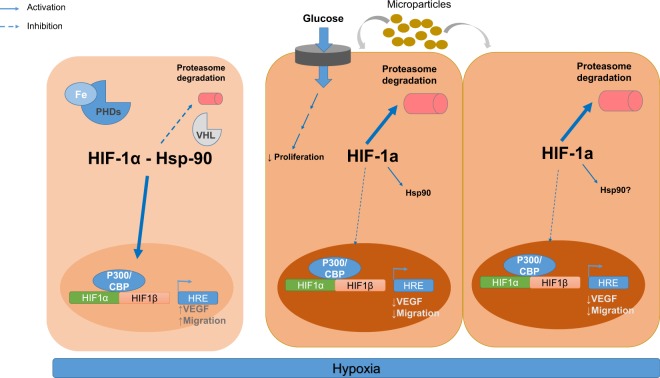
An schematic overview of the proposed mechanisms through which the hyperglycemic/hypoxic diabetic-like milieu inhibits HIF-1α-dependent responses.

Renal hypoxia and loss of tubular cells play a relevant role in diabetic kidney disease. In the diabetic milieu, the HIF-1-HRE pathway is inadequately activated, which might result in insufficient responses involved in tubular protection and repair. We report here that activation of a non-canonical, proteasome-dependent pathway of HIF-1α degradation is likely involved in the repression of the HIF-1-HRE pathway in human proximal tubular HK-2 cells exposed to HGHD-like milieu. This resulted in inhibition of VEGF-A production and cell migration, which are cell responses involved in tubular protection and repair. Strikingly, MPs generated by HK-2 cells under HGHD conditions induced all these changes in naïve HK-2 cells, which suggests that MPs may contribute to the tubular dysfunction found in DN (Fig. 9). Although it is well known that human PTC release MPs and other cell-derived vesicles which are found in urine^15,16^, to the best of our knowledge there are no previous studies on their effects on PTC themselves or on cells from distal segments along the nephron. Our current results urge further studies in this field.

Activation of the HIF system in the hypoxic diabetic kidney has been shown to be suboptimal^28–31^ or even inexistent^32^. This may explain the renoprotective effect in DN of HIF activation by CoCl_2_^32,33^ as well as the fact that a Pro582Ser polymorphism, which confers relative resistance of HIF-1 to the repressive effect of HG, is associated with DN protection^34^. Repression by HG of HIF-1α accumulation and/or HRE-dependent responses in cultured PTC under hypoxia has also been previously demonstrated in immortalized rat proximal tubular IRPTC cells^7^ and human HK-2 cells^8^. In our study, an antioxidant-insensitive, non-canonical proteasomal pathway of HIF-1α degradation was found to be responsible for the repression of the HIF-1α-HRE pathway by HG so that loss of HIF-1α stability was most likely triggered by disruption of the interaction between Hsp90 and HIF-1α, a well-known cause of non-canonical proteasomal degradation of HIF-1α^24^. Further studies in primary PTC should be done to confirm that our data can be reproduced in these cells which maintain a more differentiated phenotype than immortalized cells such as HK-2 cells.

Additional mechanisms may also contribute to the repression of the HIF-1α-HRE pathway by hyperglycemia: for instance, methylglyoxal, a glycolytic metabolite which accumulates in cells exposed to HG concentrations, has been found to trigger PHD-pVHL-independent proteasomal degradation of HIF-1α^35^ and to decrease HIF-1α transcriptional activity by impairing HIF-1 binding to the coactivator p300^29^. Another potential mechanism involved is the production of ROS: diabetes is known to be a state of high oxidative stress in kidneys. ROS production in diabetes may derive from non-enzymatic glycation, advanced glycation end products, overproduction by the mitochondria, or auto-oxidation of glucose^13^. In immortalized rat PTC α-tocopherol, an antioxidant, restored several hypoxic responses blunted by HG such as increase in the activity of an HRE-Luc transgene construct and in the transcription of HIF-regulated genes VEGF and GLUT-1^7^. However, ROS generation and expression of HIF-1α were not studied in this work. In another study in human PTC, expression of HIF-1α was unaffected by HG although HG increased ROS production^22^. In a similar way, we found here that the antioxidant NAC did not prevent the repression by HG of the accumulation of HIF-1α induced by hypoxia, even though NAC inhibited the increase in ROS production induced by the HGHD-like milieu (Fig. 2). In summary, it is unlikely that ROS production in the diabetic kidney contribute to unpaired HIF-1α regulation in HK-2 cells.

Besides studying the mechanisms involved in the repression of the HIF-1-HRE pathway in HK-2 cells, we have also analysed the consequences of this repression on cell responses such as VEGF-A production, cell migration and cell proliferation because they are involved in tubular protection and repair. Our results indicate that the three responses are inhibited by the HGHD-like microenvironment in HK-2 cells and that inhibition of VEGF-A production and cell migration may be due to repression of the HIF-1-HRE pathway (same than for the mechanisms involved in the repression of the HIF-1-HRE pathway, further studies in primary PTC should be done to confirm that our data can be reproduced in these cells). Two studies suggest that tubulointerstitial production of VEGF-A has protective role in DN through the maintenance of the peritubular capillary architecture: i) antagonism of VEGF-A signalling in db/db mice induced exacerbation of tubulointerstitial injury, which was related to loss of peritubular capillaries^36^ and ii) human renal biopsies from diabetic patients exhibited a reduction in tubulointerstitial VEGF-A expression, which was associated to diminished peritubular capillary density^9^. In addition, loss of peritubular capillaries *per se* may further aggravate tubulointerstitial hypoxia and contribute thereby to the diabetic damage of PTC with a reduced HIF-1α-dependent response to hypoxia. In connection with this, our study provides a mechanistic explanation of the repressive of effect HG on the HIF-1α-dependent production of VEGF-A in hypoxic human HK-2 cells. Regarding cell migration and proliferation, they are critical processes for renal tissue repair in DN, because tubular cell loss is one of the features of diabetic kidney disease^10^. Apoptosis has been found in the proximal tubule as well as in the distal tubule in both human and experimental DN^37–40^ and it is thought to be a key detrimental event which eventually leads to kidney injury^41^. Diabetes might affect cell migration- and cell proliferation-dependent tubular repair responses cell, as pointed out by a previous *in vivo* study^42^ and by our current results (Fig. 4b,c) results, thereby contributing to the progression of DN. However, specific *in vivo* studies should be performed to confirm this hypothesis.

Perhaps the most striking finding of the present work is that MPs from HK-2 exposed to the HGHD-like milieu are able to reproduce in control HK-2 cells all the HIF-1α-related effects of the diabetic-like microenvironment (Fig. 7). MPs, as other types of extracellular vesicles, serve as vehicles for transfer between cells of membrane and cytosolic proteins, metabolites, lipids and genetic material, such as microRNAs (miRNA), mRNAs, long noncoding RNAs and even DNA^43^. The delivery of these molecules into the recipient cells can alter their intrinsic features thereby contributing to a wide range of regulatory functions. Importantly, the composition of MPs not only depends on the parental cell type, but also on environmental factors such as high-glucose^14^ or hypoxia^44^, which may play a relevant role in the transmission through MPs of pathological changes on their target cells. Taking miRNAs as an example of the cargos contained by MPs, they have been related to the diabetic disruption of renal internal homeostasis leading to DN^45,46^. The fact that several miRNAs are HIF target genes^47^ is also particularly interesting because, given the inhibitory effect of the HGHD-like microenvironment in the activation of the HIF system in HK-2 cells, it may result in the release of MPs with a lower content in HIF-regulated miRNAs. Besides miRNAs, other molecules transported by MPs may also play a relevant role in their effects on HK-2 cells. For instance, it has been shown that MPs from patients with acute coronary syndrome decrease the interaction between eNOS and Hsp90^48^. Clearly, this effect is not dependent on RNAs, as they do not regulate protein-protein interactions. More importantly, this effect suggests that the inhibitory effect on HIF-1α up-regulation of MPs released by HK-2 cells incubated in diabetic-like milieu might be due to a decrease in the interaction between HIF-1α and Hsp-90 (i.e. the same mechanism through which high-glucose impairs the up-regulation of HIF-1α in HK-2 cells). Specific experiments are needed to confirm this hypothesis and, if confirmed, to identify the cargos contained by MPs which are responsible for the deceased interaction between HIF-1α and Hsp-90.

Hypoxia increases the release of exosomes in cancer (reviewed by Shao C *et al*^49^.) and non-cancer cells, the latter including primary PTC^50,51^. In a similar way, hypoxia also increases the release of MPs from cancer^52^ and non-cancer cultured cells such as endothelial cells^53^ and human proximal tubular HK-2 cells (the current report). Regarding high-glucose, it has been found to increase the release of exosomes or MPs in several types of cultured renal glomerular cells^27,54,55^ Given that hypoxia or high-glucose increase the release of extracellular vesicles in several types of cultured cell it is not surprising that in our experiments the incubation of PTC in HGHD conditions results in increased shedding of MPs. Unfortunately, the mechanisms involved in the increased release of MPs under hypoxia or high-glucose are still at an early stage of comprehension. In hypoxic breast cancer cells, increased release of MPs requires HIF-α dependent expression of the small GTPase RAB22A, which is a protein that localizes to budding MPs^52^. However, HIF-1-dependent mechanisms are not likely to mediate the increased release of MPs in the diabetic-like milieu because our results show that high glucose impairs the HIF-1/HRE response in hypoxia. Other mechanisms that might increase the shedding of MPs by HK-2 cells under HGHD conditions are those dependent on P2X receptors^56^ or calpains^57,58^, because they may be activated by hypoxia^59,60^, or Rho-kinase^27^ or the membrane-shaping protein caveolin-1, which may be activated by high-glucose^61^. However, as the precise mechanism of MPs release remains elusive (and it is likely to vary among different cells and depending on the stimulus for the shedding of MPs), specific experiments with inhibitors of the mentioned molecules and other signaling pathways should be performed in HK-2 cells under HGHD conditions to identify which of them are effectively involved in the increased release of MPs in the diabetic-like milieu.

As indicated above, MPs from HK-2 exposed to the HGHD-like milieu were able to reproduce in control HK-2 cells all the HIF-1α-related effects On the contrary, cell proliferation in control HK-2 cells, which was shown to be independent of HIF-1α (Fig. 4c), was unaffected by MPs from HK-2 exposed to the HGHD-like milieu. These results suggest that MPs from PTC may contribute, in a HIF-1α-dependent manner, to the tubular dysfunction found in DN. We have previously found that MPs from activated human vascular endothelial cells increase the expression of HIF-1α and the production of VEGF-A in HK-2 cells^17^. These results and the ones shown here are in good agreement with the notion that MPs may be important regulators of the expression of HIF-1α in PTC, although our current data need to be confirmed in primary PTC to give further support to this hypothesis.

In summary, our work suggests that the diabetic milieu activates in HK-2 cells a non-canonical, proteasome-dependent pathway of HIF-1α degradation which inhibits the HIF-1α-HRE pathway, either directly or through HK-2 cells -derived MPs. The resulting loss of HIF-1-dependent responses involved in PTC protection and repair might play a role in the progression of DN.

## Methods

### Reagents

Desferrioxamine, cycloheximide, propidium iodide, N- acetylcysteine, TriReagent and actinomycin D were purchased from Sigma (St. Louis, MO, USA). Mannitol was purchased from Braun (Barcelona, Spain). MG-132, PVDF membranes and Western blotting luminol reagent were purchased from Santa Cruz Biotechnology (Santa Cruz, CA, USA). 2´,7´-dichlorofluorescein diacetate (DCFH-DA) probe was purchased from Molecular Probes, (Oregon, USA). FITC (fluorescein isothiocyanate) Annexin V was purchased from BD Biosciences (Palo Alto, CA, USA). Human VEGF DuoSet was purchased from R&D Systems (Minneapolis, MN, USA). Transwells with 8-μm pore polycarbonate membrane inserts were purchased from Corning Costar (Cambridge, UK). Dynabeads™ Protein G for Immunoprecipitation was from ThermoFisher (Grand Island, NY, USA). Cell Tracer™ CM-Dil was from Invitrogen (Carlsbad, CA, USA). Antibodies were obtained from the following sources: anti-HIF-1α, anti-Hsp90 and monoclonal anti-BrdU were purchased from BD Biosciences (Palo Alto, CA, USA); anti-β-actin antibody was purchased from Santa Cruz Biotechnology (Santa Cruz, CA, USA), Goat anti-Mouse IgG (H + L) Highly Cross-Adsorbed Secondary Antibody, Alexa Fluor 488 was from ThermoFisher (Grand Island, NY, USA) and the rabbit anti-mouse IgG peroxidase conjugate antibody was from Sigma (St. Louis, MO, USA).Phalloidin CruzFluorTM 488 Conjugate was from Santa Cruz Biotechnology (Santa Cruz, CA, USA).

### Cell Culture

We used the following culture media, which were supplied by ThermoFisher (Grand Island, NY, USA): DMEM/F12, DMEM low glucose (LG, 5.5 mM glucose) and DMEM high glucose (HG, 25 mM glucose). Human renal proximal tubular epithelial cells (HK-2) were purchased from the American Type Culture Collection (ATCC) (Rockville, MD, USA). HK-2 cells were maintained in DMEM/F12 supplemented with 10% fetal bovine serum (FBS), 1% penicillin/streptomycin/amphoterycin B, 1% glutamine and 1% insulin-transferrine-selenium (ITS) (ThermoFisher, Grand Island, NY, USA). Renal cell carcinoma cell line (RCC4) was maintained in DMEM HG supplemented as above. One week before beginning the experiments cell culture media were changed to DMEM LG (ThermoFisher, Grand Island, NY, USA) supplemented as above. Cells were routinely maintained in 5% CO_2_ (normoxic conditions) at 37 °C. For experiments involving MPs, media were previously filtrated (0.2 μM pore size) and, for characterization of MPs released by PTC, serum MPs were eliminated by ultracentrifugation (100.000 x g, for 60 min).

In all experiments, cells were plated at 70–90% confluence and when completely attached, they were exposed to either medium DMEM LG or DMEM HG supplemented with 0.5% FBS, 1% penicillin/streptomycin/amphoterycin B, 1% glutamine and 1% ITS under hypoxic (1% oxygen) or normoxic conditions (21% oxygen). Hypoxia experiments were performed in an In vivo200 hypoxia workstation (Ruskinn Technology, West Yorkshire, UK). Cells were manipulated and lysed inside the chamber, and all media and buffers were pre-equilibrated to 1% O_2_.

### Western blot analysis

Cells were split into six-well plates at a density of 3 × 10^5^ cells/well. Immediately after treatments, cells were washed with ice-cold phosphate-buffered saline (PBS) and scraped into ice-cold passive lysis buffer (Promega, Madison, WI, USA) in the presence of a cocktail of protease inhibitors (Roche, Basel, Switzerland). Cells were kept on ice for 30 min, and pelleted by centrifugation at 4000 × g, for 5 min, at 4 °C. Proteins from cell lysates were first denatured by heating and then resolved onto 8% SDS-polyacrylamide gels. The resolved proteins were transferred to a PVDF membrane for 1 h, in 50 mM Tris-HCl, 380 mM glycine, 0.1% SDS and 20% methanol. Blots were incubated overnight at 4 °C with mouse anti-HIF-1α (1:1000) or mouse hsp-90 (1:2000) antibodies. After incubation for 1 h at room temperature with the corresponding secondary antiserum (1:4000), the signal was detected w*i*th western blotting luminol reagent using β-actin antibody (1:5000) as loading control.

### Immunofluorescence analysis

HK-2 cells (10^4^ cells/glass coverslip) were treated as indicated in the results section. HK-2 cells were fixed with 2% paraformaldehyde for 10 min, permeabilized with 0.1% (v/v) Triton X-100, washed with PBS, blocked with 4% bovine serum albumin (BSA) for 1 h at room temperature and incubated overnight at 4 °C with anti-HIF-1α (1:100 dilution) antibody. Cells were then incubated at 37 °C with α-mouse-Alexa-Fluor® 488 (1:1000) for 1 h in the darkness. Coverslips were then washed and mounted with ProLong Gold antifade reagent with DAPI (Invitrogen Eugene, OR, USA). Detection was performed by using a Leica SP5 confocal microscope (Leica Microsystems, Wetzlar, Germany), through the Confocal Microscopy Service (ICTS ‘NANBIOSIS’ U17) of the Biomedical Research Networking Centre on Bioengineering, Biomaterials and Nanomedicine (CIBER-BBN at the University of Alcalá, Madrid, Spain). HIF-1α-dependent immunofluorescence intensity was quantified after digital capture using image-J software.

### Immunoprecipitation assay

HK-2 cells were seeded in cell culture dishes (100 mm diameter) one day before experiments. After the different treatments, cells were lysed in 500 μl RIPA buffer (Santa Cruz Biotechnology, Santa Cruz, CA, USA) with protease inhibitor cocktail. Cell lysates (1 mg of protein) were centrifuged (4,000xg, for 10 min) and supernatants were transferred into fresh tubes. Then, 2 μg of antibodies anti-HIF-1α were added and incubated overnight at 4 °C. Thereafter, 20 μl of Dynabeads™ Protein G for Immunoprecipitation were added and incubated at 4 °C for an additional 3 h period. Beads were collected, washed three times with 500 μl PBS and finally supplemented with 30 μl 2 × SDS-PAGE sample buffer and boiled at 95 °C, for 5 min. Beads were removed by centrifugation and supernatants were loaded onto 8% SDS-PAGE. Western blot analysis was performed as above.

### Luciferase assay

Cells were split into p35 plates at a density of 4 × 10^5^ cells/plate 24 h before transfection. Cells were then incubated for 5 h at 37 °C with 1 ml OptiMEM (Invitrogen, CA, USA) containing complexes of 5 μL lipofectamine 2000 (Invitrogen, CA, USA), 1 μg human p9HIF1-Luc firefly luciferase reporter plasmid [it contains nine copies of the human HIF binding sequence located between positions −958 and −951 of the 5′ human VEGF gene promoter and was generously gifted by Dr. Manuel Ortiz de Landázuri, (Service of Immunology, Hospital Princesa, Madrid, Spain)] and 0.5 μg renilla luciferase reporter pRL-CMV as an internal control. Transfected cells were next incubated under hypoxia for 16 h in either LG medium or HG medium. Finally, firefly luciferase activity was measured with a Lumat LB9506 luminometer (Berthold Technologies, Herts, UK) and normalized against renilla luciferase activity by using the dual-luciferase reporter assay system (Promega, Madison, WI, USA).

### Transient transfection with siRNA

For HIF-1α inhibition we used HIF-1α siRNA (ThermoFisher, Grand Island, NY, USA) and silencer negative control siRNA (ThermoFisher, Grand Island, NY, USA) as a control. HK-2 cells at 90% of confluence were transfected with 100 nM HIF-1α-siRNA, or 100 nM silencer negative control siRNA using Lipofectamine 2000 reagent (ThermoFisher, Grand Island, NY, USA). 24 h after transfection, cells were used for the experiments.

### Trans-well migration assays

Cell migration was determined using Trans-well chambers with 8-μm sized pores (Corning Costar, Cambridge, UK). HK-2 cells (250.000 cells/0.1 mL) were placed in the upper chambers of each insert cultured in either HG medium or LG medium in the absence of FBS and ITS, whereas the lower chambers were filled with HG medium or LG medium supplemented with 10% FBS and 1% ITS. After 24 h of incubation under hypoxia conditions (1% O_2_) with or without MPs, the inserts were removed and fixed with methanol for 8 min. Cells on the top chamber were eliminated. Cells adhering to the bottom of the Transwell membrane were stained with 0.1% crystal violet. Three representative fields of each Trans-well were captured using a microscopy. (Optika, Ponteranica, Italy). Quantitative analysis of migrated cells was performed in a blind manner by manual count in three randomly selected fields and results were expressed as a percentage of migrated cells vs control

### Isolation RNA, RT, PCR and qPCR

Total cell RNA was isolated with TriReagent. One microgram of total RNA was reverse-transcribed using 200 U NZY M-MuLV reverse transcriptase (Nzytech, Lisbon, Portugal) in the buffer supplied with the enzyme supplemented with 50 µM oligo dT, 40 U NZY Ribonuclease Inhibitor (Nzytech, Lisbon, Portugal), and 0.5 mM of deoxyribonucleotides (dNTPs). RT-conditions were a preincubation at 25 °C for 10 min and then at 37 °C for 50 min; the reaction was inactivated by heating at 70 °C for 15 min. cDNA product was stored at −20 °C until used.

Two microliters of cDNA were then PCR-amplified with specific primers for HIF-1α sense: 5′- GAAAGCGCAAGTCCTCAAAG-3′, antisense: 3′- TGGGTAGGAGATGGAGATGC-5′ and for VEGF_165_ sense: 5′-ATCTTCAAGCCATCCTGTGTGC-3′ and antisense: 3′-TCACCGCCTCGGCTTGTCACCAT-5′ using NZYtaq DNA polymerase (Nzytech, Lisbon, Portugal) according to the instruction of the manufacturer. The signals were normalized with the β-actin gene expression level; the primers for β-actin were: sense 5′-AGAAGGATTCCTATGTGGGCG-3′ and antisense: 5′-CATGTCGTCCCAGTTGGTGAC-3′. PCR-conditions were: denaturation at 94 °C for 5 min, followed by 34 cycles of 95 °C for 1 min, 57 °C, for 1 min, 72 °C for 1 min, and then a final cycle of 10 min at 72 °C. The PCR products were separated by electrophoresis and visualized in 2% agarose gels.

Quantitative PCR analysis was performed using Taqman gene expression (Applied Biosystems, Foster City, CA, USA) according to the instruction of the manufacturer. 0.1 µg cDNA was used for each sample and were amplified and quantified by TaqMan probes (Applied Biosystems, Foster City, CA, USA): HIF-1α (#Hs00936368m1). Results of real-time PCR determinations were presented as Ct values, where Ct was defined as the threshold cycle number at which product is first detected by fluorescence. The amount of target was normalized with the endogenous reference, gene GADPH (#Hs03929097g1). ΔCt was the difference in Ct values derived from the corresponding gene and GADPH gene in each sample assayed. ΔΔCt represented the difference between paired samples. The n-fold differential ratio was expressed as 2^−ΔΔCt^. Equal efficiency of all the primers was previously confirmed.

### Determination of VEGF secretion

HK-2 cells were placed in 24 well plates (75 × 10^3^ cells/well) for 24 h. Controls received only medium. After the different treatments, the medium was removed and kept at −80 °C for ELISA assays. VEGF was analyzed using human VEGF DuoSet (R&D Systems, Minneapolis, MN, USA). VEGF mouse capture antibody diluted in phosphate-buffered saline (PBS) (pH 7.4) was incubated onto each well of a 96-well plate overnight (RT). Plates were washed three times between each step with PBS-Tween (0.05%). After blocking with bovine serum albumin (1%) in PBS for 1 h (RT), recombinant human VEGF standards diluted in blocking solution, or protein sample was added to each well. After incubation, 100 μl of biotinylated goat anti-human VEGF was added to each well, and plates were left for 2 h. Streptavidin-HRP 1:200 diluted in PBS was added and plates were incubated at room temperature for 20 min. Then, a H_2_O_2_ and tetramethylbenzidine (1:1) solution was added, protected from light and incubated for 20 min at room temperature. The reaction was stopped with 2 NH_2_SO_3_ and absorbance was immediately read at 450 nm. Samples were analysed by triplicated^62^.

### Cell proliferation assay with 5′-Br-2′-deoxyuridine (BrdU)

DNA synthesis was assessed by BrdU uptake. HK-2 cells were placed in 24-well plates (40 × 10^3^ cells/well). Cells were treated with LG medium or HG medium for 24 h under hypoxic (1% O_2_) or normoxic conditions. Cells were then pulsed with 10 μM BrdU (BD Bioscience. Palo Alto, CA, USA) during the last 1 h of incubation. Afterwards, the cells were fixed with 2% paraformaldehyde, for 15 min. Cells were washed with PBS and DNA was partially denatured by incubation with 2 M HCl for 20 min at room temperature. The reaction was stopped by incubation for 2 min with 0.1 M Na_2_B_4_O_7_. Cells were then washed three times with PBS containing 0.05% Tween-20 (pH 7.4) and 0.1% BSA. Finally, cells were sequentially incubated in the dark with antibodies against BrdU (1:50; 1 h) and anti-mouse Alexa Fluor 488 (1:1000; 1 h). Coverslips were then washed and mounted with ProLong Gold antifade reagent with DAPI. Detection was performed by confocal laser scan microscopy LEICA TCS-SL (Heidelberg, Germany). Quantitative analysis of BrdU-positive cells was performed in a blind manner by manual count in five randomly selected fields of BrdU-positive nuclei, as compared to total cell count per field (total cells were visualized as DAPI-positive nuclei).

### Measurement of reactive oxygen species (ROS) generation

The relative levels of ROS were assessed with the fluoroprobe DCFH-DA. In order to avoid hypoxia/reoxygenation-induced ROS production, cells from treatments involving hypoxia were maintained in hypoxic conditions along all the procedures required for ROS analysis. In order to determine ROS, HK-2 cells in suspension were incubated for 45 min, at 37 °C, with DCFH-DA and propidium iodide was added before flow cytometry analysis to identify living cells. Afterwards cellular fluorescence intensity was measured by flow cytometry with a FACSCalibur cytometer (Becton Dickinson, USA)

### Microparticle isolation and characterization

HK-2 grown in LG medium were incubated 24 h under the four experimental conditions (i.e. LG or HG and normoxia or hypoxia; they were ≈ 2 × 10^6^ cells cultured in 10 mL medium at the end of the 24 h incubation). Media were collected and cleared from detached cells and cells fragments by centrifugation at 500 x g for 5 min. Subsequently, MPs were pelleted by centrifugation of the resulting supernatant at 18,000 x g for 20 min. Unless otherwise indicated, MPs were washed with PBS and finally resuspended in LG medium supplemented with 0.5% FBS, 1% penicillin/streptomycin/amphoterycin B, 1% glutamine and 1% ITS. MPs were immediately used for experiments.

Four different approaches were used for MPs characterization: i) Protein content: it was measured using the Pierce BCA-200 Protein Assay Kit (ThermoFisher, Grand Island, NY, USA) according to the manufacturer’s instructions. ii) Flow cytometry. Gallios flow cytometer (Beckman-Coulter, USA) was calibrated with Megamix-Plus fluorescent beads (Biocytex, Marseille, France) according to the manufacturer’s instructions. MPs were considered as events within the gates fixed with Megamix-Plus beads and positive for Annexin V iii) Nanoparticle tracking analysis (NTA): The enumeration of isolated MPs by NTA was undertaken on a NS300 NTA machine (Malvern, Amesbury, United Kingdom). MPs were diluted in PBS and their particle size and distribution were analyzed at recommended standard conditions. At least three videos with 60 s, with more than 500 tracks per video, were taken per sample iv) Transmission Electron Microscopy (TEM) analysis: Samples were observed in negative staining mode, using a copper grid covered by a “holey film” carbon layer and the contrast staining was performed with a uranyl acetate solution 1% w/v. Grids were viewed using a FEI Tecnai G2 Spirit (FEI Europe, Eindhoven, Netherlands) and photographed with Olympus digital camera (Soft Image Solutions GmbH, Germany).

Internalization of MPs into cells was studied in the following way: HK-2 cells under hypoxia were incubated with Cell Tracer™ in order to label MPs produced by them. Fluorescently prelabelled MPs (red) were then isolated and added to recipient HK-2 cells whose cell membrane was stained with phalloidin (green). Internalization of MPs into cells was checked by confocal microscopy.

### Statistical analysis

Each experiment was repeated three times including the indicated replicates. The results are expressed as the mean ± SD. They were subjected to one way analysis of variance (ANOVA) following by the Bonferroni’s test for multiple comparisons. The level of significance was set at P ≤ 0.05.
