# Author Correction: Mechanism and Consequences of The Impaired Hif-1α Response to Hypoxia in Human Proximal Tubular HK-2 Cells Exposed to High Glucose

**DOI:** 10.1038/s41598-020-65511-1

**Published:** 2020-05-20

**Authors:** Coral García-Pastor, Selma Benito-Martínez, Victoria Moreno-Manzano, Ana B. Fernández-Martínez, Francisco Javier Lucio-Cazaña

**Affiliations:** 1 Departamento de Biología de Sistemas, Universidad de Alcalá, Alcalá de Henares, Madrid, Spain; 2 Neuronal and Tissue Regeneration Laboratory, Centro de Investigación Príncipe Felipe, Valencia, Spain; 3 Departamento de Biología, Universidad Autónoma de Madrid, Madrid, Spain

Correction to: *Scientific Reports* 10.1038/s41598-019-52310-6, published online 01 November 2019

This Article contains errors.

As a result of figure assembly error, in Figure 4B the LG hypoxia image duplicates the LG normoxia image. The correct Figure 4B is included below as Figure 1.

**Figure 1:**
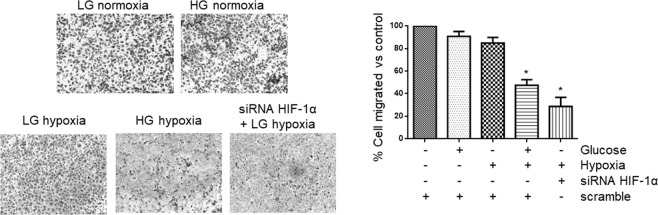
.

In addition, delineation is missing for some blots in Figure 3C and 7A. The corrected Figures 3C and 7A is included below as Figure 2 and Figure 3, respectively.

**Figure 2:**
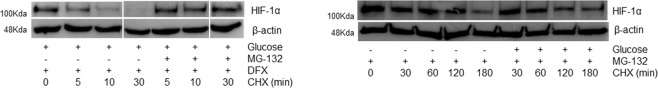
.

**Figure 3:**
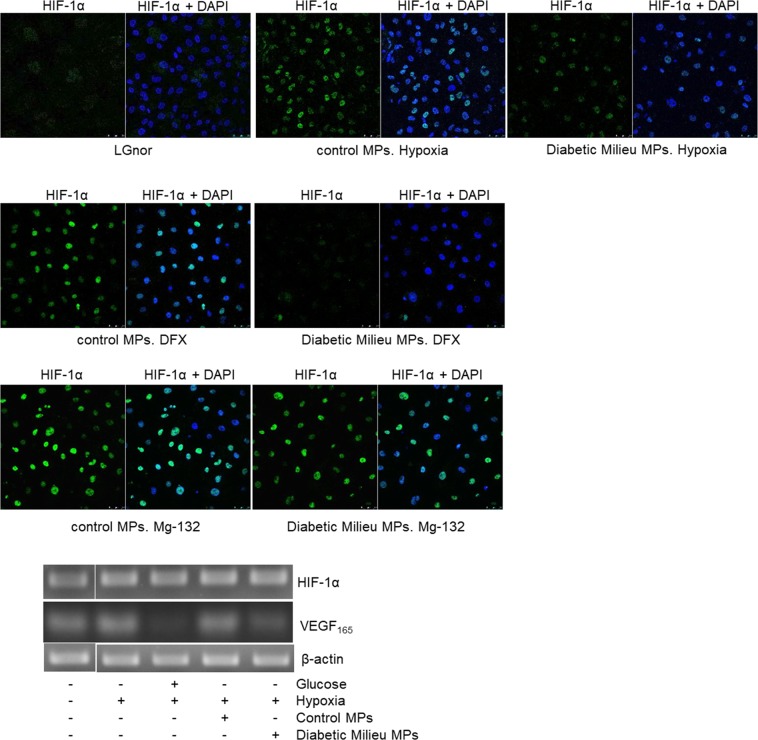
.

Finally, a supplementary file containing images of raw data, which was included with the initial submission, was omitted from the original version of this Article. This file appears below.

## Supplementary Information

Supplementary Information.

