# Supplementary material for: Author Correction: Mechanism and Consequences of The Impaired Hif-1α Response to Hypoxia in Human Proximal Tubular HK-2 Cells Exposed to High Glucose
Source: Sci Rep. 2020 May 20;10:8642. doi: 10.1038/s41598-020-65511-1 (PMC7237441; doi:10.1038/s41598-020-65511-1)
Supplement: Supplementary file 1 — Supplementary Information. [file 41598_2020_65511_MOESM1_ESM.pdf]

MECHANISM AND CONSEQUENCES OF THE IMPAIRED HIF-1α RESPONSE TO HYPOXIA IN HUMAN PROXIMAL TUBULAR HK-2 CELLS EXPOSED TO HIGH GLUCOSE

Coral García-Pastor[1*,a], Selma Benito Martínez[1,b], Victoria Moreno-Manzano[2,c], Ana B. Fernández-Martínez[3, #, d], Javier Lucio-Cazana[1, #,e]

[1]Departamento de Biología de Sistemas, Universidad de Alcalá, Alcalá de Henares, Madrid

[2] Neuronal and Tissue Regeneration Laboratory, Centro de Investigación Príncipe Felipe, Valencia, Spain.

[3]Departamento de Biología, Universidad Autónoma de Madrid, Madrid

RUNNING TITLE: Impaired HIF-1α regulation in diabetic-like milieu

Fig 1

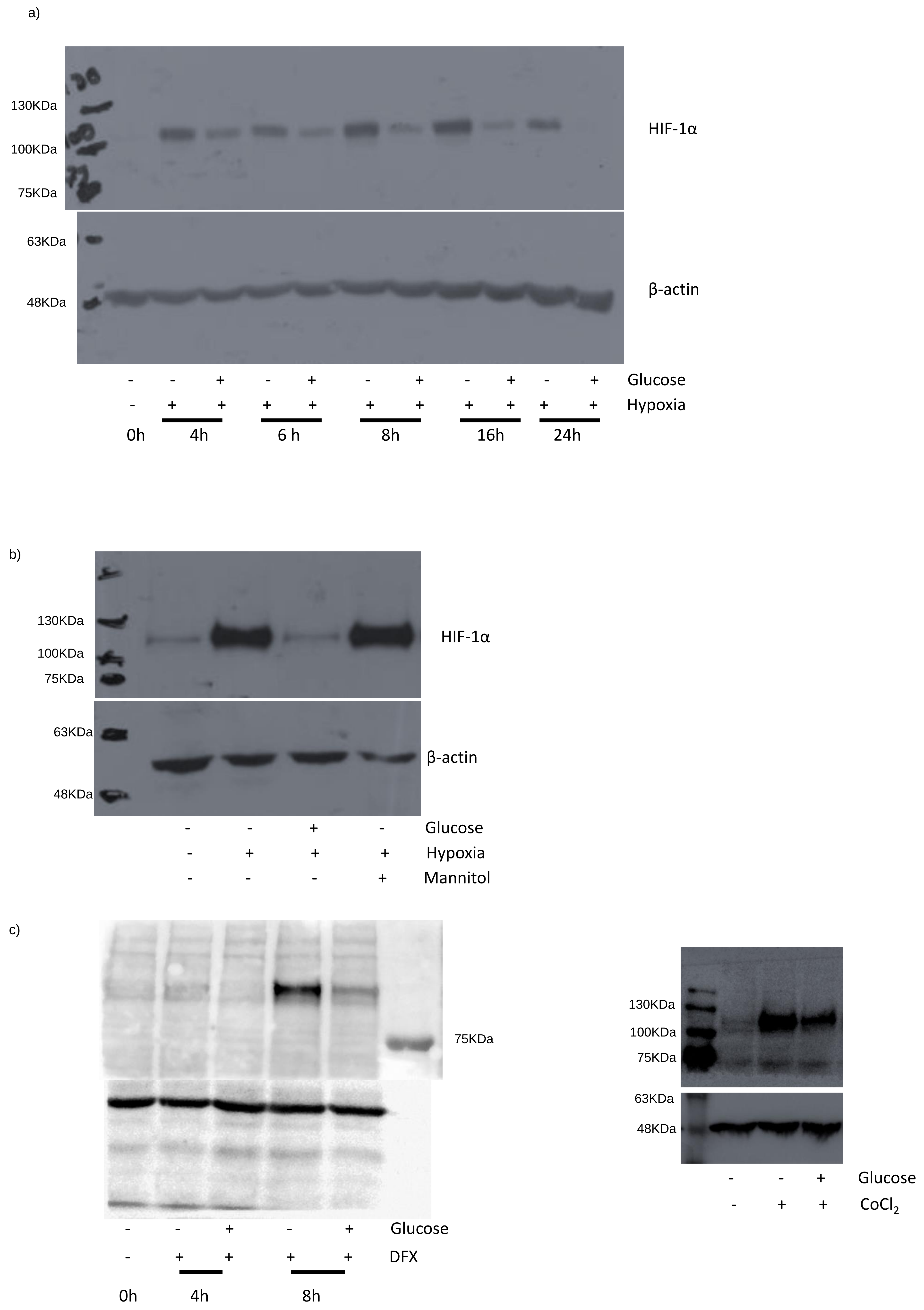

a)
130KDa
100KDa
75KDa
63KDa
48KDa
HIF-1α
β-actin
- - + - + - + - + - + Glucose
- + + + + + + + + + + Hypoxia
0h 4h 6 h 8h 16h 24h
b)
130KDa
100KDa
75KDa
63KDa
48KDa
HIF-1α
β-actin
- - + - Glucose
- + + + Hypoxia
- - - + Mannitol
c)
75KDa
- - + - + Glucose
- + + + + DFX
0h 4h 8h
130KDa
100KDa
75KDa
63KDa
48KDa
- - + Glucose
- + + CoCl2

Fig 2

b)

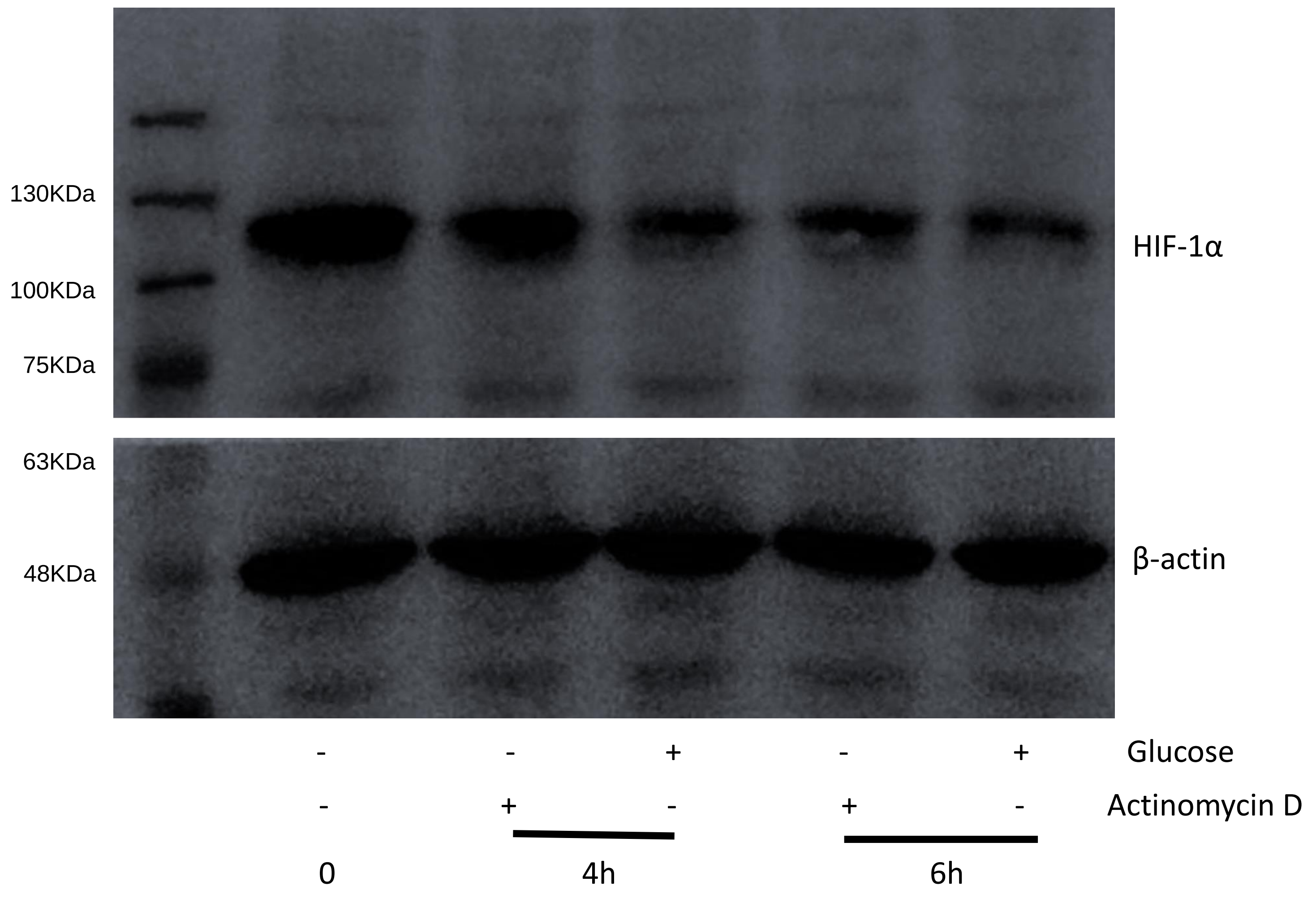
130KDa
100KDa
75KDa
63KDa
48KDa
HIF-1α
β-actin
- - + - + Glucose
- + - + - Actinomycin D
0
4h
6h

d)

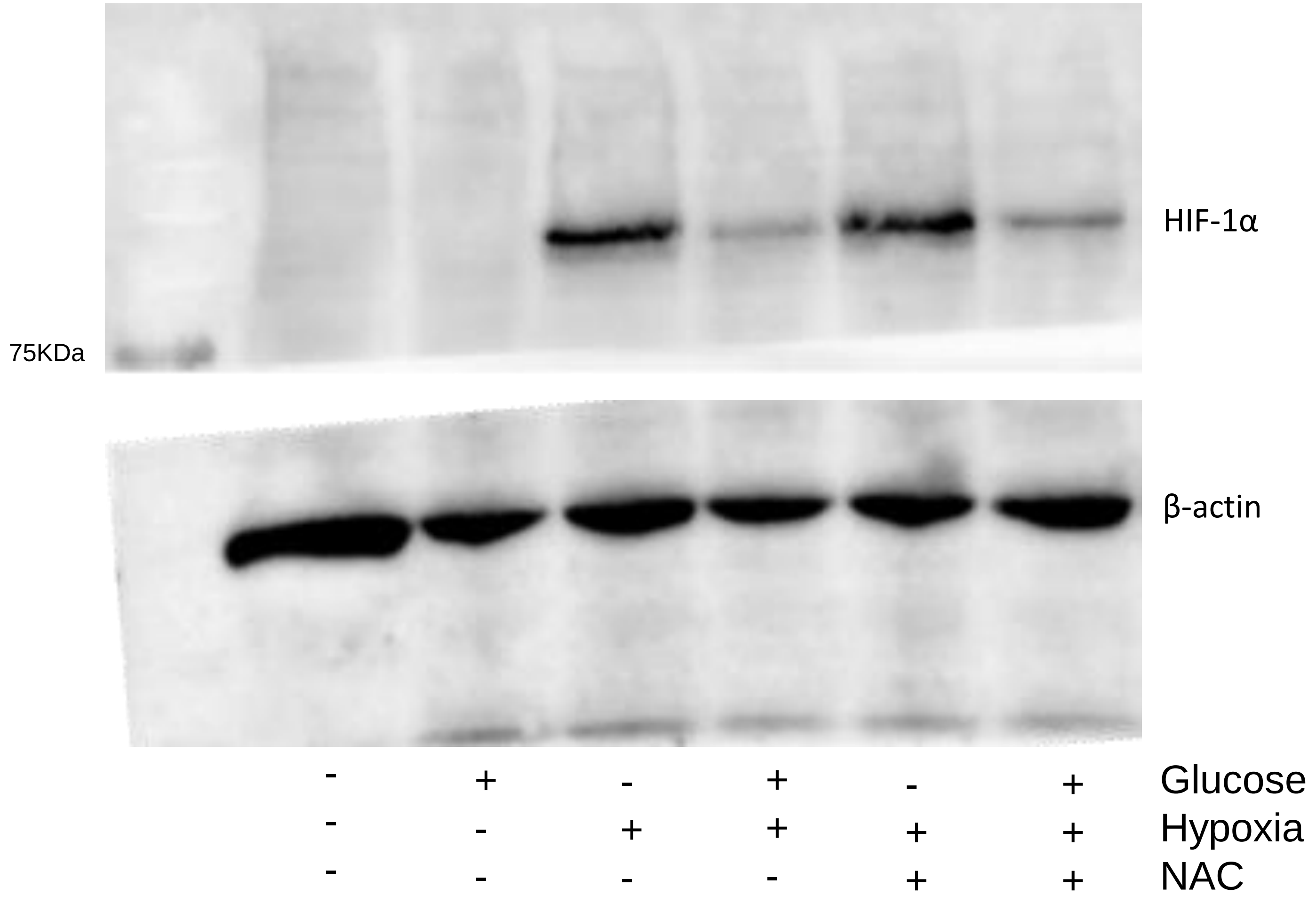
75KDa
HIF-1α
β-actin
- + - + - + Glucose
- - + + + + Hypoxia
- - - - + + NAC

Fig 3

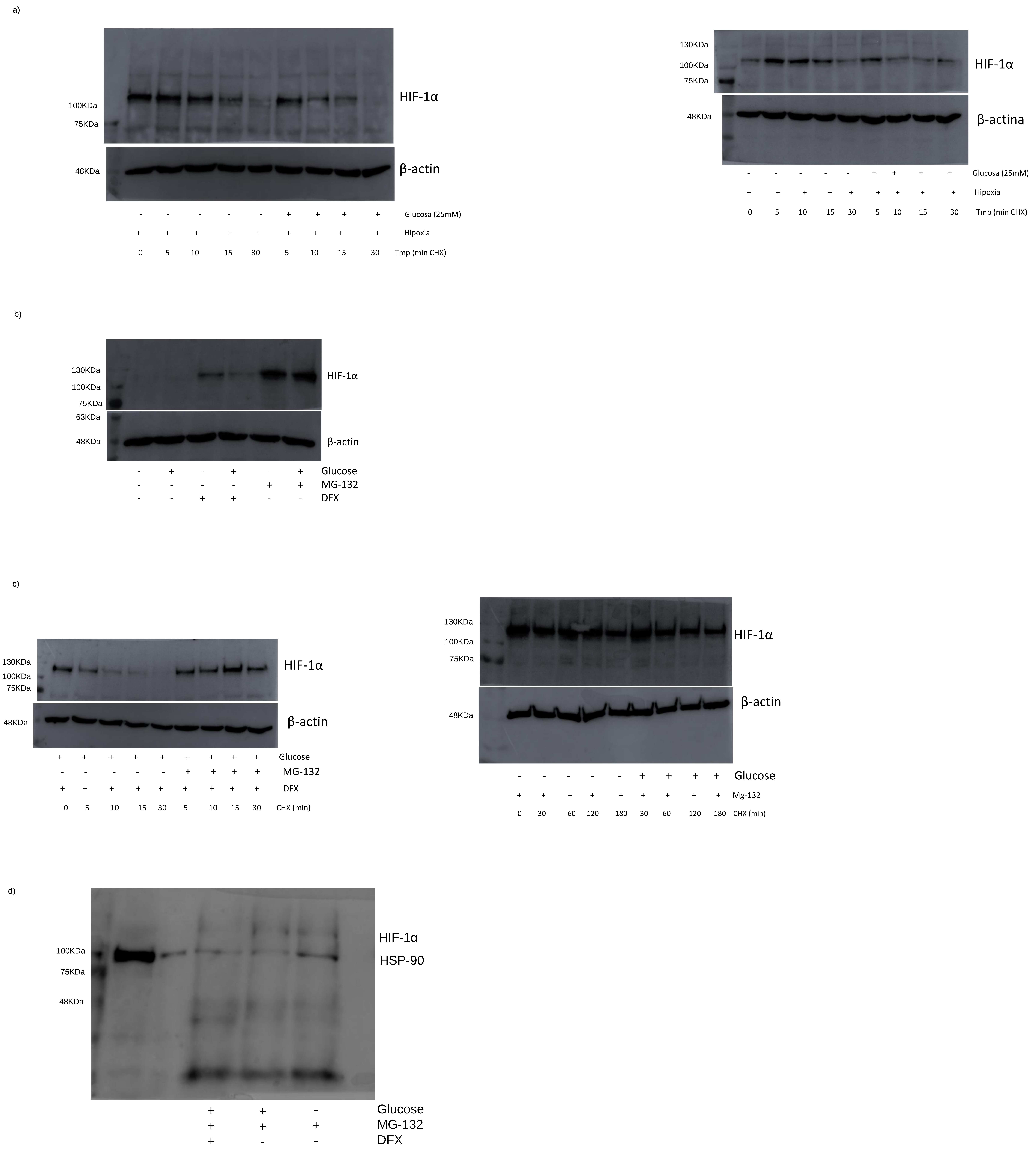
a)
100KDa
75KDa
HIF-1α
48KDa
β-actin
- - - - - + + + + Glucosa (25mM)
+ + + + + + + + + Hipoxia
0 5 10 15 30 5 10 15 30 Tmp (min CHX)
130KDa
100KDa
75KDa
HIF-1α
48KDa
β-actina
- - - - - + + + + Glucosa (25mM)
+ + + + + + + + + Hipoxia
0 5 10 15 30 5 10 15 30 Tmp (min CHX)
b)
130KDa
100KDa
75KDa
63KDa
48KDa
HIF-1α
β-actin
- + - + - + Glucose
- - - - + + MG-132
- - + + - - DFX
c)
130KDa
100KDa
75KDa
HIF-1α
48KDa
β-actin
+ + + + + + + + + Glucose
- - - - - + + + + MG-132
+ + + + + + + + + DFX
0 5 10 15 30 5 10 15 30 CHX (min)
130KDa
100KDa
75KDa
HIF-1α
48KDa
β-actin
- - - - - + + + + Glucose
+ + + + + + + + + Mg-132
0 30 60 120 180 30 60 120 180 CHX (min)
d)
100KDa
75KDa
48KDa
HIF-1α
HSP-90
+ + - Glucose
+ + + MG-132
+ - - DFX

Fig. 4

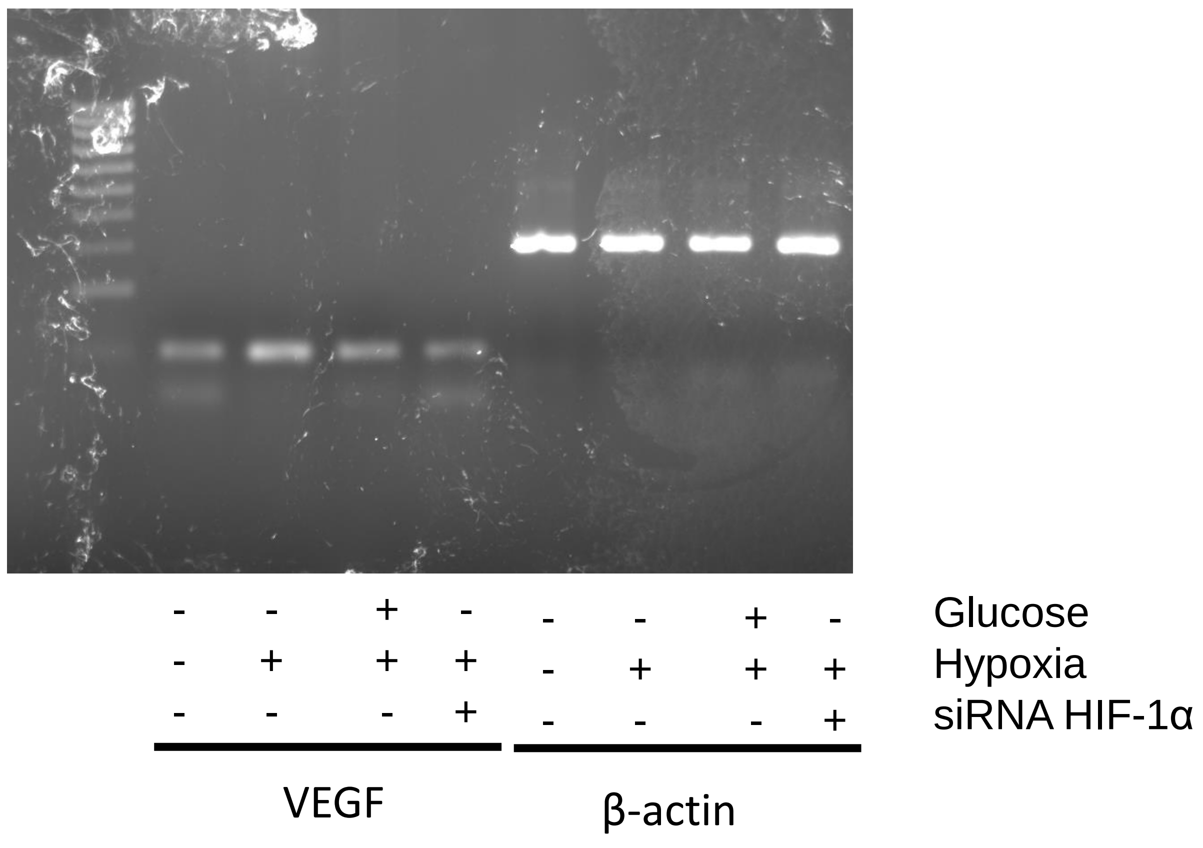

Fig. 7a

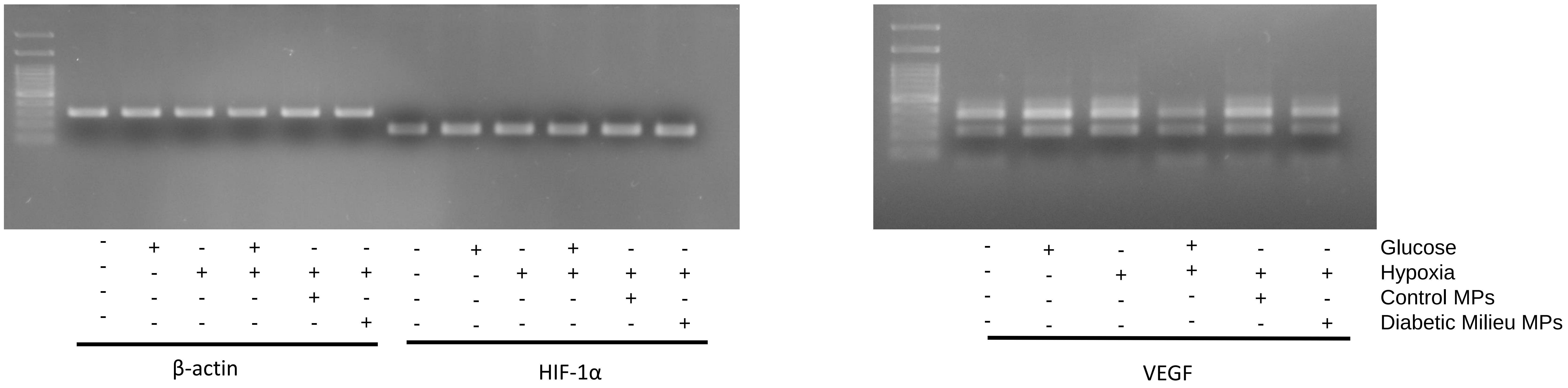

Glucose
Hypoxia
Control MPs
Diabetic Milieu MPs
β-actin
HIF-1α
VEGF
